# Reliability and validity of the EQ-5D-3L for Kashin–Beck disease in China

**DOI:** 10.1186/s40064-016-3613-3

**Published:** 2016-11-07

**Authors:** Hua Fang, Umer Farooq, Dimiao Wang, Fangfang Yu, Mohammad Imran Younus, Xiong Guo

**Affiliations:** 1 School of Nursing, Health Science Center of Xi’an Jiaotong University, Xi’an, Shaanxi China; 2 Institute of Endemic Diseases, School of Public Health, Health Science Center of Xi’an Jiaotong University, Xi’an, Shaanxi China; 3 Department of Community Medicine, Ayub Medical College, Abbottabad, Pakistan

**Keywords:** EQ-5D, Health-related quality of life, Kashin–Beck disease, Reliability, Validity

## Abstract

**Background:**

Kashin–Beck Disease (KBD) is an endemic osteoarthropathy in areas which extend from the North-East to the South-West of China. Most of the patients with KBD suffer multiple dysfunctions in major joints causing decreased health status. However because of their low education level and unique living habits, it is hard to find tools to measure the health-related quality of life (HRQOL). European quality of life (EQ-5D-3L) patient-reported instrument is widely used to measure HRQOL. This study aimed to establish the validity and reliability of the Chinese version of the EQ-5D-3L for evaluating HRQOL of KBD individuals in rural area.

**Methods:**

368 individuals who were suffering from KBD were recruited through stratified multistage random sampling from Shaanxi province, China. The EQ-5D-3L and the WHOQOL-BREF were administrated in each individual by face to face interview. Test–retest reliability was assessed at 10–14 days intervals. The test–retest reliability was measured by calculating the Kappa coefficients for EQ-5D-3L five dimensions. For the EQ VAS, the intraclass correlation coefficient (ICC) was computed. Convergent and divergent analysis, construct validity was established using Spearman’s rank correlation between the EQ-5D-3L and the WHOQOL-BREF. Known groups’ validity was examined by comparing groups with a priori expected differences in health-related quality of life (HRQOL).

**Results:**

For 362 individuals (98%), comprehensive data of all the EQ-5D-3L dimensions were available. Kappa values of the EQ-5D-3L five items ranged from 0.324 to 0.554. ICC of the EQ VAS was 0.497. For convergent validity, the three items (self-care, usual activity, and mobility) of EQ-5D-3L, index scores, and VAS showed moderate correlations with the physical health domain of the WHOQOL-BREF (r absolute value ranged from 0.339 to 0.475). For divergent validity, the 5 items of EQ-5D-3L showed weak or no correlations with environment and social relationship domains of WHOQOL-BREF. The Chinese EQ-5D-3L clearly demarcated between groups which were reporting severe disease degree, poorer general health, more number of painful joints with worse HRQOL.

**Conclusions:**

The EQ-5D-3L Chinese Version demonstrated fair to moderate levels of test–retest reliability and adequate construct validity in KBD individuals in China.

## Background

Kashin–Beck Disease (KBD) is an endemic osteoarthropathy with chronic damage to the involved joints, cartilage, bone marrow and skeletal muscle which results in skeletal deformation, pain and dysfunction (Guo 2001; Cao et al. 2008; Schepman et al. 2011). The pathology of KBD is still unknown. In the past 150 years, multiple environmental risk factors have been postulated to cause the disease. Recently a model comprised of an interaction between genetic and environmental factors has been hypothesized with the major evidence pointing to the environmental factors such as deficiency of selenium and contamination of cereals (Guo et al. 2014).

Typically, the disease is initiated in childhood, and presents itself as fixed flexion of the terminal joint with or without radiological changes in the fingers mostly metaphyseal area. This results in enlarged painful joints, restricted mobility, shortening of upper and lower limbs. The more severe form of disease may result in dwarfism (Guo 2001).

KBD is endemic in areas which extend from the North-East to the South-West of China (Sokoloff 1988; Hinsenkamp 2001). There are 0.65 million KBD patients in 378 KBD endemic counties having a total population of 38.07 million (National Health and Family Planning Commission of People’s Republic of China National Health and Family Planning Commission of People’s Republic of China 2014). This disease is most prevalent in the provinces of Shaanxi, Sichuan, Tibet, and Qinghai (Chinese Kashin–Beck Disease Surveillance Group 2003, 2006). The KBD endemic areas are mostly rural with low socio economic status of the population (Hinsenkamp 2001). Further, more than 40% KBD individuals have no school education (Fang et al. 2012) and the annual household income is much lower than the general population in urban China (Wang et al. 2012). Most of the patients suffer multiple dysfunctions in major joints causing decreased health status (Guo 2001; Schepman et al. 2011). However because of their low education level and unique living habits, it is hard to find tools to measure the health-related quality of life (HRQOL). Fang et al. (2012) developed a KBD specific instrument for assessment of HRQOL.

European quality of life (EQ-5D-3L) patient-reported instrument is widely used to measure HRQOL. It is one of the most frequently used generic tools for assessing the HRQOL in skeletal and muscular disorders (Picavet and Hoeymans 2004; Lin et al. 2014; Alava et al. 2013). The Chinese language EQ-5D-3L was validated for the population of China in urban areas (Wang et al. 2012), but most KBD individuals have low education level and live in rural areas. Previous studies measuring HRQOL of KBD utilizing the instrument did not report the reliability of the KBD (Farooq et al. 2010).

This study aimed to establish the validity and reliability of the Chinese version of the EQ-5D-3L for evaluating HRQOL of KBD individuals in rural area.

## Methods

### Sample and study design

Stratified multi-stage random sampling was done in Shaanxi province of China. It is one of the most endemic KBD areas of China (Chinese Kashin–Beck Disease Surveillance Group 2003, 2006). Two counties were randomly selected from 30 most prevalent KBD counties of Shaanxi province, and then six villages were randomly selected from two counties, three villages from each county. The diagnosis and degree of severity of KBD were assessed based on the standard KBD diagnostic criteria: an individual who has resided in a KBD endemic area for at least 6 months showing the following clinical or radiological changes. (1) Radiological changes in the distal end of the bones of the middle and proximal phalanges of the index and ring fingers. (2) Focal or irregular premature closure of the epiphysis. (3) Limited motion and enlargement of peripheral joints, deformities and dwarfism. (4) Involvement of multiple joints by non-inflammatory lesions (Guo 2001). The KBD was divided three degrees according to severity of disease, higher degree means more severity. The criteria of 1st degree are enlarged finger joints, arthritic pain in the knee and ankle joints, and abnormal X-ray signs (metaphysical lesions in phalange). The 2nd degree KBD patients exhibit shortened fingers and clinical symptoms of 1st degree and worse X-ray signs than 1st degree patients. The criteria of 3rd degree are retarded growth or dwarfism and clinical symptoms of 1st and 2nd degree and worse X-ray signs than 2nd degree.

The individuals were diagnosed by a physician with expertise in the diagnosis of KBD. Inclusion criteria were: diagnosis of KBD and greater than 18 years of age. Exclusion criteria were: inability of understanding questionnaires. Questionnaires included the EQ-5D-3L (Chinese version) (EuroQol Group 2013), WHOQOL-BREF (WHOQOL group 1996) and a general profile questionnaire. Because more than 70.0% of individuals with KBD had low educational level of primary or lower (Fang et al. 2012). They were not able to read the questionnaires. Thus, this study was conducted by face to face interview. The interview was performed using Mandarin (Chinese) at local community hospital. The interviewer filled the general profile questionnaire then explained the WHOQOL-BREF and EQ-5D to the respondents and asked them to fill in the WHOQOL-BREF, EQ-5D and VAS.

The interviewers were graduate students of the department of public health of Xi’an Jiaotong University and staff of the department of public health of local community hospital. They were trained by the researchers.

For assessing test–retest reliability (Streiner and Norman 2008), a second interview was performed at the same place by the same interviewers 10–14 days after the first interview. Before performing retest, all individuals were asked if there was any change in their health status since the last assessment. Those who reported any change were excluded from retest.

The Ethics review Committee of Xi’an Jiaotong University School of Medicine approved the study. A thorough explanation of the study was provided to potential subjects. Informed consent was acquired in writing from all the participating individuals.

### Instruments

The general questionnaire included information about the socio-demographic profile, KBD degree, painful joints and the general health item with response options ‘very good’, ‘good’, ‘fair’, ‘poor’, ‘very poor’.

The EQ-5D-3L (EuroQol Group 2013) consists of five dimensions with three-level options (EQ-5D-3L) and EQ VAS (visual analogue scale). The five dimensions include Pain/Discomfort, Self-care, Usual Activities, Mobility and Anxiety/Depression. Scores for the five dimensions, by applying EQ-5D-3L value sets, can be transformed into index scores that range from −0.149 to 1, higher score means better QOL. China EQ-5D-3L value sets were used in this study. It generated using the time trade-off method and published in 2014 (Liu et al. 2014). Before 2014, UK, Japan or USA EQ-5D-3Lvalue sets were most applied as a substitute (Wang et al. 2012; Cao et al. 2012; Jin et al. 2012).

WHOQOL-BREF is a validated instrument for Chinese population (Fang et al. 1999; Power et al. 2006). It contains a total of 26 questions with four domains (psychological health, physical health, social relationships, and environmental) and two general items (G1 and G4) which inquire about overall perception of an individual’s own QOL or health (WHOQOL group 1996). The four domain scores denote an individual’s perception of quality of life in each particular domain. Domain scores range from 0 to 20, and are scaled in a positive direction (i.e. higher scores denote higher quality of life). Domain scores were used to analyze data from this scale.

### Statistical analysis

Validity and Reliability of the EQ-5D-3L were evaluated using established guidelines (Scientific Advisory Committee of the Medical Outcomes Trust 2002).

### Test–retest reliability

The reliability of test–retest was measured by calculating the Kappa coefficients (Cohen 1968) for EQ-5D-3L five dimensions between test and retest response and percentages of agreement. Kappa value 0.81–1.0 indicates an almost perfect agreement, 0.61–0.80 substantial agreement, 0.41–0.60 moderate agreement, 0.21–0.40 fair agreement, below 0.20 indicates a slight agreement (Landis and Koch 1977).

For the EQ VAS, the intraclass correlation coefficient (ICC) (Fleiss and Cohen 1973) was computed. An ICC of more than 0.80 indicated excellent reproducibility, and one between 0.61 and 0.80 moderate reproducibility and one between 0.41 and 0.60 fair reproducibility (Shrout 1998).

### Convergent and divergent validity

Convergent and divergent validity were examined by the correlations between EQ-5D-3L dimensions, EQ VAS and previously validated WHOQOL-BREF using Spearman’s rank correlation. We expected that comparable dimensions would correlate better than less comparable dimensions. Because four items of the EQ-5D (mobility, usual activity, self-care, and pain/discomfort) are chiefly correlated with the physical domain of the WHOQOL-BREF, the anxiety dimension of EQ-5D is chiefly correlated with the psychological domain of the WHOQOL-BREF, we expected the following: (1) The four items (self-care, usual activity, mobility, and pain/discomfort) of EQ-5D-3L, index scores, and VAS would show moderate to high correlations with the physical health domain of the WHOQOL-BREF as convergent; (2) The anxiety of EQ-5D-3L would show moderate to high correlations with the psychological domain of the WHOQOL-BREF as convergent; and (3) Because five items of the EQ-5D are not directly correlated with the environment and social relationships domain of the WHOQOL-BREF, we expected the 5 items of EQ-5D-3L, index scores, and VAS would show weak or no correlations with this two domains as divergent.

Spearman’s rank correlation coefficient of 0.5 or above indicates high correlation, 0.30–0.49 moderate correlation, and lowers than 0.30 weak correlation (Cohen 1988).

### Known groups’ validity

The validity of identified groups’ was evaluated by comparing the EQ-5D-3L index scores and EQ VAS for subgroup with different KBD degree, the general health and number of painful joints using Kruskal–Wallis tests. We expected individuals with more severe disease, poorer general health, and a greater number of painful joints would have lower EQ-5D-3L index scores and EQ VAS scores.

All data analysis was carried out using SPSS16.0. *P* values <0.05 were considered statistically significant.

## Results

### Sample characteristics

368 individuals with KBD were selected from 6 villages which were in disease endemic areas of Shaanxi province. Six individuals were omitted because they did not complete EQ-5D-3L. Additional two individuals did not complete WHOQOL-BREF. The socio-demographic characteristics and related clinical features of the 362 KBD patients are listed in Table 1. The sample had an average age of 56.1 years (SD 10). The majority of the individuals were farmers (n = 349, 96.4%), with an educational level of primary or lower (n = 260, 71.9%). Individuals available for retesting were 95.

**Table 1:** Socio-demographic and clinical features

	First test (%) n = 362	Retest (%) n = 95
*Gender*		
Male	177 (48.9)	44 (46.3)
Female	185 (51.1)	51 (53.7)
*Age (years)*		
Mean ± SD	56.1 ± 10.0	58.3 ± 10.9
Age–range	28.0–80.0	28.0–80.0
*Marital status*		
Married	313 (86.5)	82 (86.3)
Widow	41 (11.4)	10 (10.5)
Single	8 (2.2)	3 (3.2)
*Profession*		
Farmer	349 (96.4)	90 (94.7)
Others	13 (3.6)	5 (5.3)
*Educational level*		
No schooling	145 (40.1)	37 (38.9)
Primary school	115 (31.8)	32 (33.7)
Middle school	80 (22.1)	20 (21.1)
High school and higher	22 (6.1)	6 (6.3)
*KBD degree* ^a^		
1st degree	223 (61.6)	62 (65.3)
2nd degree	125 (34.5)	32 (33.7)
3rd degree	14 (3.9)	1 (1.1)

^a^Higher KBD degree, worse health status

### EQ-5D-3L response

The range of reporting no problems varied between 21% for pain and discomfort to 56.6% for self-care (Table 2a). No obvious ceiling effects were observed. The mean of EQ VAS was 60.59 (SD 18.31, range 0–100) (Table 2b). The mean of index score was 0.644 (SD 0.232, range −0.149 to 1) (Table 2b).

**Table 2:** EQ-5D-3L responses and Scores based on China value sets

EQ-5D-3L	N	No problem (%)	Some problem (%)	Extreme problem (%)
Mobility	362	120 (33.1)	238 (65.7)	4 (1.1)
Self-care	362	205 (56.6)	148 (40.9)	9 (2.5)
Usual activity	362	157 (43.4)	198 (54.7)	7 (1.9)
Pain/discomfort	362	76 (21.0)	232 (64.1)	54 (14.9)
Anxiety	362	143 (39.5)	179 (49.4)	40 (11.0)

	N	Minimum	Maximum	Mean	S.D.
*EQ VAS*	362	0	100	60.59	18.306
Index score based on China value sets	362	−0.149	1.00	0.644	0.232
*WHOQOL-BREF*					
Physical score	360	4.00	17.71	11.137	2.518
Psychology score	360	5.33	16.67	12.290	2.325
Social score	360	4.00	16.00	14.220	2.312
Environment score	360	5.50	16.00	11.911	1.851

### Test–retest reliability

The retest was performed in 95 KBD individuals. Before performing retest, all 368 individuals were asked if there was a change in the status of physical or mental health as compared to earlier test. Individuals who reported any change were omitted from the retest. The time interval of test–retest was 10–14 days. Table 3 showed the results on test– retest reliability. The test–retest agreement ranged from 65 to 81% and Kappa coefficients ranged from 0.324 to 0.554 in 5 dimensions of EQ-5D-3L. For the EQ VAS, the ICC was 0.497.

**Table 3:** Test–retest reliability of EQ-5D-3L (n = 95)

EQ-5D-3L	Agreement between test and retest	Kappa coefficient
Mobility	77 (81%)	0.554
Self-care	63 (66%)	0.338
Usual activity	62(65%)	0.324
Pain	68 (72%)	0.400
Anxiety	71 (75%)	0.552

### Convergent and divergent validity

Table 4 showed convergent and divergent validity. As expected, stronger correlation coefficients were observed between EQ-5D-3L and WHOQOL-BREF for comparable dimensions as compared to those observed between less comparable dimensions. (1) The four items (self-care, usual activity, mobility, and pain/discomfort) of EQ-5D-3L, index scores, and VAS showed moderate correlations with the physical health domain of the WHOQOL-BREF (r absolute value ranged from 0.339 to 0.475) except EQ-5D-3L pain dimension (r = −0.269). (2) The anxiety of EQ-5D-3L showed weak correlations with the psychological domain of the WHOQOL-BREF (r = −0.286). (3) The 5 items of EQ-5D-3L, index scores, and VAS showed weak or no correlations with environment domain of the WHOQOL-BREF (r absolute value ranged from 0.049 to 0.209), The 5 items of EQ-5D-3L showed weak correlations with social relationship domain of the WHOQOL-BREF (r absolute value ranged from 0.159 to 0.240) except EQ-5D-3L self-care dimension (r = −0.337). While index scores, and VAS showed moderate correlations with social relationship domain of the WHOQOL-BREF (r = −0.307, −0.356, respectively).

**Table 4:** Correlations (Spearman’s rho) between the EQ-5D-3L and WHOQOL-BREF, n = 360

	EQ-5D mobility	EQ-5D self-care	EQ-5D usual activites	EQ-5D pain	EQ-5D anxiety	EQ VAS	Index score
WHOQOL-BREF Physical health	−0.414**	−0.386**	−0.339**	−0.269**	−0.444**	0.475**	0.454^**^
WHOQOL-BREF Psychological	−0.262**	−0.341**	−0.300**	−0.138**	−0.286**	0.343**	0.333^**^
WHOQOL-BREF Social relationships	−0.191**	−0.337**	−0.240**	−0.159**	−0.228**	0.356**	0.307^**^
WHOQOL-BREF Environment	−0.114*	−0.192**	−0.209**	−0.049	−0.152**	0.148**	0.177^**^

** P < 0.05, * P < 0.01

### Known groups’ validity

As expected, individuals reporting poor general health status, showed significantly lower scores of EQ VAS and EQ-5D-3L index, individuals who reported more number of painful joints had lower EQ-5D-3L index scores and VAS scores, severer disease degree with low EQ-5D-3L index scores and EQ VAS score (Table 5).

**Table 5:** EQ VAS and EQ-5D-3L index scores comparison for individuals with differing health condition (N = 362)

Health condition	N	EQ-5D index		EQ VAS	
		Mean	SD	Mean	SD
*Degree*					
1st degree	223	0.664	0.240	61.31	18.517
2nd degree	125	0.630	0.199	60.63	16.405
3rd degree	14	0.419	0.266	43.50	24.153
*P*		<0.01		<0.01	
*Self-reported overall health*					
1 good/very good	62	0.825	0.217	74.94	11.902
2 fair	71	0.694	0.169	68.96	13.155
3 poor	190	0.607	0.215	57.03	16.404
4 very poor	39	0.443	0.213	39.00	17.963
*P*		<0.01		<0.01	
*Number of painful joints*					
0	53	0.748	0.230	70.25	11.460
1–3	145	0.654	0.243	61.68	18.745
4–8	164	0.600	0.212	56.01	18.494
*P*		<0.01		<0.01	

## Discussion

To the best of our knowledge, the current study is the first to evaluate validity and reliability of Chinese version of the EQ-5D-3L in KBD individuals. The results showed that the EQ-5D-3L had good response, fair-moderate test–retest reliability and acceptable construct validity.

### Response

The response rate was 98%, and completion rate was 100% indicating good feasibility of the EQ-5D-3L in KBD individuals. Table 2a showed high rates of reporting problems. The health problem that was reported the most by the sample was pain/discomfort followed by problems with mobility. These findings correspond with the clinical manifestation of KBD. Most individuals with KBD have deformed and painful lower extremities that affect mobility (Guo 2001; Schepman et al. 2011). The health problem that was reported the least was self-care. This may be because KBD takes place in childhood and these individuals learn how to live with their disability as they grow older. Lower ceiling effects in KBD individuals were observed than in general population in urban China (Wang et al. 2012).

### Test–retest reliability

Table 3 showed fair-moderate levels of test–retest reliability in the EQ-5D-3L 5 items, while the VAS showed fair test–retest reliability. It could be explained by the low ability of KBD individuals understanding the VAS. Because 40% individuals had no education and an additional 32% had only a primary education (Table 1), these individuals may have had difficulty understanding what the questions were asking. During the face to face interview, more than 30% individuals thought the VAS was difficult to understand. This indicates that there is a need for further modification of VAS instructions and its use in an aging population with limited education in rural China.

### Convergent and divergent validity

For convergent validity, the 4 items (self-care, usual activity, mobility, and pain/discomfort), index score, and VAS score of EQ-5D-3L had greater correlation coefficients with physical domain than other domains of the WHOQOL-BREF, and the correlations were moderate as we expected except pain. The correlation between pain of the EQ-5D and physical domain of the WHOQOL-BREF should be further studied in the future. One possible reason is that some items of the physical domain do not correlate with pain. The correlation between anxiety of the EQ-5D and psychological domain of the WHOQOL-BREF was weak. This finding was different from our expectation. The correlation between anxiety of the EQ-5D and psychological domain of the WHOQOL-BREF should be further studied in the future.

For divergent validity, the 4 items (mobility, usual activity, pain/discomfort and anxiety) of EQ-5D-3L showed weak or no correlations with environment and social relationship domains of WHOQOL-BREF as we expected. These results supported our hypotheses. However, we found that self-care, index scores, and VAS of EQ-5D-3L showed moderate correlations with the social relationship domain of the WHOQOL-BREF (r = −0.337, −0.307, −0.356, respectively). These findings are similar to previous studies showing that small—moderate correlation existed between the index score/VAS scores of EQ-5D social relationship domain (Hung et al. 2015; Konig et al. 2007). The findings were somewhat different from our hypotheses. A possible reason is that better self-care ability results in less dependence on other’s help and higher satisfaction with social relationships.

### Known groups’ validity

Validity of the EQ-5D-3L for the identified groups was established using known groups’ analysis. Individuals who reported more severe KBD, poorer general health, and more painful joints had lower EQ-5D-3L index scores and EQ VAS score.

### Limitation

There were some limitations in this study. First, we recruited individuals with KBD from only the Shaanxi province in China which limits the generalizability of our results to individuals who are similar to this sample. Second, we focused only on the validity and reliability of the EQ-5D-3L. Further study should be conducted to measure responsiveness of Chinese version EQ-5D-3L in individuals with KBD.

## Conclusion

The EQ-5D-3L Chinese Version demonstrated fair to moderate levels of test–retest reliability and adequate construct validity in KBD individuals in China. Having a reliable and valid health-related quality of life instrument, the EQ-5D-3L, may elucidate the quality of life issues and health care needs for aging persons with KBD living in rural China.
